# Choroid vascular occlusion and ischemic optic neuropathy after facial calcium hydroxyapatite injection- a case report

**DOI:** 10.1186/s12893-015-0007-3

**Published:** 2015-03-08

**Authors:** Chien-Chih Chou, Hsin-Han Chen, Yi-Yu Tsai, You-Ling Li, Hui-Ju Lin

**Affiliations:** Department of Ophthalmology, China Medical University Hospital, No. 2 Yuh Der Road, Taichung, 404 Taiwan; Department of Plastic and Reconstructive Surgery, China Medical University Hospital, Taichung, Taiwan; Department of Medical Research, China Medical University Hospital, Taichung, Taiwan; School of Chinese Medicine, College of Chinese Medicine, China Medical University, Taichung, Taiwan

**Keywords:** Calcium hydroxyapatite, Nose augmentation, Choroid vascular occlusion, Ischemic optic neuropathy, Vision loss, Filler

## Abstract

**Background:**

We reported a case of sudden monocular vision loss after calcium hydroxyapatite (CaHA) injection into the nasal tip and dorsum with detailed retina images.

**Case presentation:**

A healthy, 35-year-old woman received CaHA filler injection for nose augmentation. Ten minutes after the procedure, she developed nausea, vomiting, headache, ptosis, and left periorbital pain. After 30 minutes, she complained of progressively blurring vision in the left eye. The best-corrected visual acuity (BCVA) in her left eye was 30 cm ahead of hand motion. Left exotropia was noted in primary gaze. Limitations in adduction, supraduction, and infraduction of the left eye were also observed. Slit lamp examination of the left eye revealed a pink conjunctiva, a clear cornea, a mild anterior chamber reaction, a sluggish papillary light reflex, and a semi-dilated pupil. A positive relative afferent pupillary defect was observed in the left eye. Fundus examination revealed optic disc edema and some linear whitish opacity over the superior and temporal sites in the left eye, suggesting multiple CaHA emboli in the choroid vessels.

**Conclusions:**

Although the majority of adverse reactions are mild and transient, surgeons should be alert about extremely rare serious adverse events such as visual loss.

## Background

Facial plastic surgery reverses the signs of aging. Injectable facial fillers are effective in ameliorating certain signs of aging [1], and calcium hydroxyapatite (CaHA) is one of the most commonly used fillers for this purpose [2]. Serious adverse events are rarely observed, and the majority of adverse reactions are mild and transient [3]. Here we report the case of a 35-year-old woman who developed sudden monocular vision loss after CaHA injection into the nasal tip and dorsum.

## Case presentation

**Figure 1:**
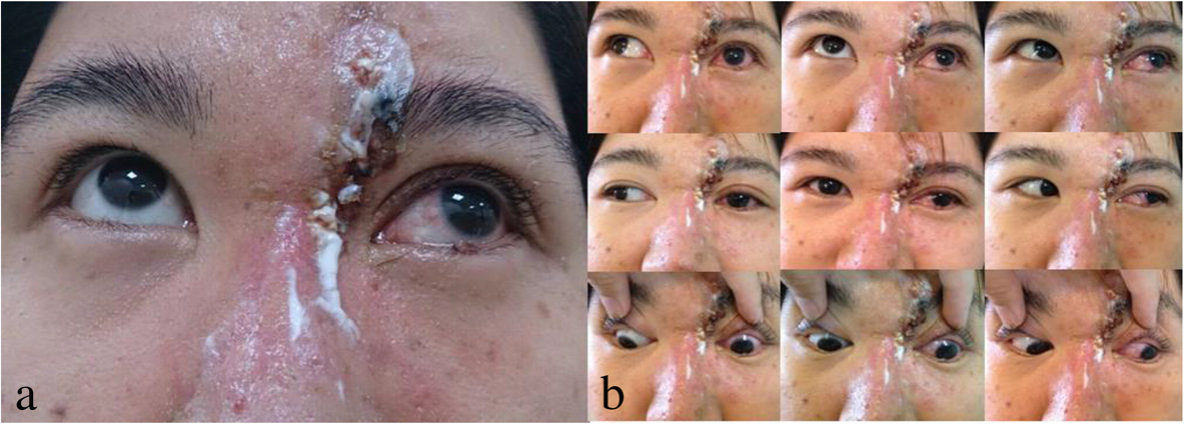
**Skin necrosis developed at nasal dorsum, glabellar region, and left forehead (a).** Left exotropia was noted in primary gaze, and limitations on adduction, supraduction, and infraduction in left eye were also noted **(b)**.

**Figure 2:**
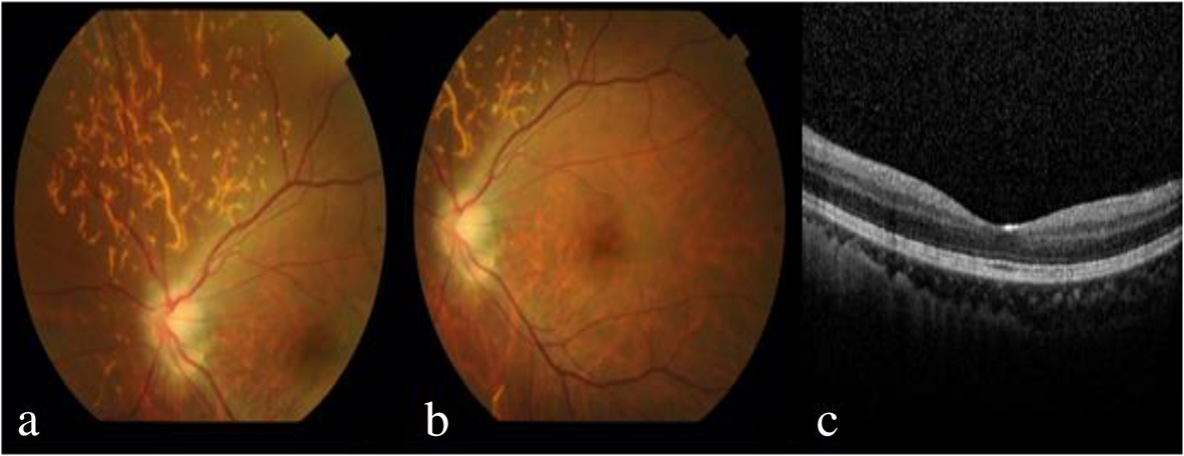
**Fundus examination revealed optic disc edema and some linear whitish opacities over the superior and temporal sites in the left eye, suggesting multiple CaHA emboli in the choroid vessels (a).** No macular edema in left eye was revealed on fundus examination **(b)**, or OCT **(c)**.

**Figure 3:**
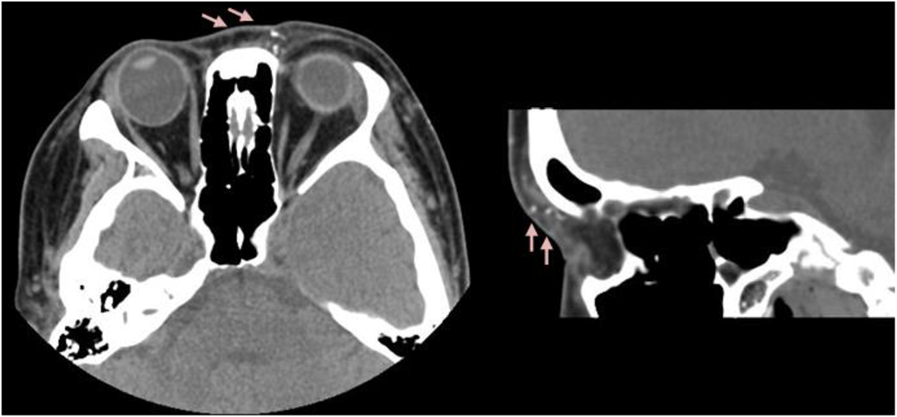
**Orbital CT demonstrates multiple radiopaque spots in the subcutaneous layer of medial aspect of left periorbital region, suggesting CaHA deposition.**

A healthy, 35-year-old woman without any history of ocular and systemic disease received CaHA filler injections (RADIESSE® 1.5 ml) for cosmetic nose augmentation. Multiple injections along midline of the nasal dorsum from nasal tip to glabella were performed under local anesthesia. Ten minutes after the procedure, she developed nausea, vomiting, headache, ptosis, and left periorbital pain. After 30 minutes, she complained of progressively blurring vision in the left eye. The best-corrected visual acuity (BCVA) in her left eye was 30 cm ahead of hand motion. Skin necrosis developed over the nasal dorsum, glabellar region, and left forehead (Figure 1a). Left exotropia was noted in primary gaze. Limitations in adduction, supraduction, and infraduction of the left eye were also observed (Figure 1b). Slit lamp examination of the left eye revealed a pink conjunctiva, a clear cornea, a mild anterior chamber reaction, a sluggish pupillary light reflex, and a semi-dilated pupil. A positive relative afferent pupillary defect was observed in the left eye. The intraocular pressure was normal in both eyes. Fundus examination revealed optic disc edema and some linear whitish opacities over the superior and temporal sites in the left eye, suggesting multiple CaHA emboli in the choroid vessels (Figure 2a,b). Optical coherence tomography (OCT) revealed disc edema without macular edema in the left eye (Figure 2c). Fluorescein angiography revealed neither delayed filling nor hypofluroescence in the left eye. Visual field testing revealed an inferior altitudinal visual field defect in the left eye. Measurement of the visual evoked potential (VEP) showed a decreased amplitude and marked delay in the appearance of peaks. Electroretinography (ERG) showed a normal waveform. All examinations were normal in the right eye. Orbital computed tomography (CT) demonstrated high-density deposits in the nose region and left medial orbital cavity (Figure 3). No evident lesion was noted on brain magnetic resonance imaging (MRI).

Alprostadil and dextran were administered for improving blood supply. Moreover, ten sessions of hyperbaric oxygen therapy were administered. One month later, the visual acuity in her left eye improved to 6/60. A pale disc was observed, with persistent plaque occlusions in the choroid vessels.

## Discussion

There are few reports of vision loss following facial injection of autologous fat or hyaluronic acid [4-6]. In a previous study, Lazzeri et al. [7] reported 32 cases of blindness caused by iatrogenic retinal embolism after cosmetic facial filler injections, while Park et al. [8] reported 12 cases of retinal artery occlusion caused by cosmetic facial filler injections. Furthermore, Park et al. [9] showed the clinical and angiographic features of occlusion of the ophthalmic artery and its branches caused by cosmetic facial filler injections. In these 44 cases, only one was attributed to CaHA, and no CaHA emboli were clearly observed on fundus photography.

Sung et al. [10] postulated that emboli may move in a retrograde fashion to the ophthalmic artery under a high injection pressure. In the present case, multiple emboli localized on the choroidal layer without retinal vessel occlusion, resulting in normal ERG waveform. However, poor vision, a positive RAPD sign, and a pale, swollen disc were present. Visual field testing showed an inferior altitudinal visual field defect. We postulate that the CaHA emboli migrated via the dorsonasal artery back to the main ciliary arteries and occluded the short posterior ciliary arteries, which supply the superior nasal choroid and the optic nerve. Subsequently, ischemic optic neuropathy developed and caused poor vision, a positive RAPD sign, a pale, swollen disc, and an abnormal waveform on VEP. Furthermore, we first postulated that the occluded vessel was Haller’s layer because the distribution pattern of affected vessels was consistent with the Haller’s layer distribution pattern.

The emboli moved back to branches supplying the oculomotor nerve, causing blepheroptosis and ophtalmoplegia. This is compatible with the CT findings (Figure 3). The ptosis and limitation in supraduction subsided gradually. We postulate that the superior division of the oculomotor nerve innervating the levator and superior rectus muscles recovered early.

In previously reported cases of CaHA injections and in this case, there was no cerebral infarction, which is more frequently observed after autologous fat injections [9]. This may be related to properties of the filler material, such as molecular weight or size.

To decrease the risk of intravascular injection and retrograde occlusion, Park et al. suggested slow injection in a fractionated dose and the use of a blunt cannula [9]. We suggest the use of a mixture of CaHA and epinephrine because epinephrine leads to vasoconstriction and thus decreases the possibility of intravascular injection. Soft tissue can be dissected to create a subcutaneous space for subsequent filler injection. Furthermore, an injection device with a valve that can relieve excessive injection pressure can be designed for this purpose.

Injectable facial fillers have become increasingly popular these days. Although the majority of adverse reactions are mild and transient, surgeons should be alert about extremely rare serious adverse events such as visual loss. Most cases of blindness are caused by autologous fat and hyaluronic acid injections. To the best of our knowledge, localized choroid vascular occlusion, ischemic optic neuropathy, and cranial nerve III palsy without evidence of compromised retinal or choroidal circulation after CaHA injection have not been reported.

## Conclusion

In conclusion, we reported a case of sudden monocular vision loss after CaHA filler injection into the nasal tip and dorsum. CaHA emboli were clearly observed in this case, providing direct evidence to prove the mechanism underlying retrograde occlusion after facial filler injection.

### Consent statement

Written informed consent was obtained from the patient for publication of this case report and any accompanying images. A copy of the written consent is available for review by the Editor of this journal.
